# Rapid and recoverable in vivo magnetic resonance imaging of the adult zebrafish at 7T

**DOI:** 10.1016/j.mri.2016.10.013

**Published:** 2017-04

**Authors:** Gavin D. Merrifield, James Mullin, Lindsay Gallagher, Carl Tucker, Maurits A. Jansen, Martin Denvir, William M. Holmes

**Affiliations:** a Glasgow Experimental MRI Centre, Institute of Neuroscience and Psychology, University of Glasgow, UK; b University/BHF Centre for Cardiovascular Science, University of Edinburgh, UK

**Keywords:** Zebrafish, MRI, Solenoid, In vivo, Flow cell, Physiological monitoring

## Abstract

Increasing scientific interest in the zebrafish as a model organism across a range of biomedical and biological research areas raises the need for the development of in vivo imaging tools appropriate to this subject. Development of the embryonic and early stage forms of the subject can currently be assessed using optical based techniques due to the transparent nature of the species at these early stages. However this is not an option during the juvenile and adult stages when the subjects become opaque. Magnetic resonance imaging (MRI) techniques would allow for the longitudinal and non-invasive assessment of development and health in these later life stages. However, the small size of the zebrafish and its aquatic environment represent considerable challenges for the technique. We have developed a suitable flow cell system that incorporates a dedicated MRI imaging coil to solve these challenges. The system maintains and monitors a zebrafish during a scan and allows for it to be fully recovered. The imaging properties of this system compare well with those of other preclinical MRI coils used in rodent models. This enables the rapid acquisition of MRI data which are comparable in terms of quality and acquisition time. This would allow the many unique opportunities of the zebrafish as a model organism to be combined with the benefits of non-invasive MRI.

## 1 Introduction

The zebrafish (*Danio rerio*) has rapidly emerged as an important model organism for scientific research across a variety of biological and biomedical fields [1], [2], [3]. Traditionally, mammalian model organisms, e.g. rodents, have been pre-eminent, largely due to the homology of mammalian genomes, anatomy, cell biology and physiology. Nevertheless Howe et al. [4] have highlighted the significant parallels between the human and zebrafish genomes. Furthermore, the zebrafish is an emerging model system, with many additional practical advantages over mammalian models. These include a short life cycle, simplicity of large-scale breeding and low maintenance costs. These make large scale studies far more timely and economic than is possible with other vertebrate models. The zebrafish also has unique features of interest uncommon in most other vertebrate species, such as natural tissue regenerative capabilities. This combination has proven to be extremely attractive to a wide range of bio-scientists, ranging from evolutionary biology to regenerative medicine. The optical transparency of the zebrafish embryo has enabled its widespread use to study early development by serial in vivo imaging using standard microscopy techniques. However, there are currently no imaging modalities deployed that can successfully image the optically opaque post-embryonic life stages in vivo. At the same time there is growing interest in using adult zebrafish as models of inherited and acquired human disease [1], [5], [6].

While many non-optical imaging techniques (such as X-rays, ultrasound and magnetic resonance imaging (MRI) etc) have been successfully deployed in mammalian research, the small size of the adult zebrafish (typically <35 mm in length) and physiological requirements of the subject have precluded these imaging modalities from practical use with zebrafish. The aquatic habitat of the zebrafish is also a particular problem not typically encountered or accommodated for by these modalities. Indeed, MRI can introduce additional difficulties for both the imaging aspects of a study and with regard to experimental practicalities. There have been previous examples of in vivo NMR spectroscopy in larger fish e.g. tilapia (*Oreochromis mossambicus*) [7], Crassius X [8] and Atlantic cod [9], as well as MRI studies of large fish e.g. carp [10] and eelpout [11]. In contrast, there has been limited use of MRI techniques in the much smaller (~2 cm) and bio-medically important zebrafish [12], [13], [14], [15], [16]. Previous studies, however, have generally used existing MRI hardware with a minimal focus on physiological maintenance or subject welfare.

However, if in vivo MRI could be deployed more rigorously in the adult zebrafish it could provide a powerful imaging research tool for a broad range of biological research activities. Furthermore, the non-invasive longitudinal imaging provided by MRI, with its inherent soft-tissue contrast and versatility, could provide novel scientific avenues, rapid throughout and economic advantages in zebrafish research. In seeking to address this opportunity we have developed a novel MRI coil and animal handling system, optimized for high resolution scanning of the in vivo adult zebrafish under physiological conditions. In this paper, we describe this system and present examples of MRI images acquired with this system highlighting its unique research potential.

## 2 Materials and methods

### 2.1 Zebrafish application and ethical review

All experiments were approved by The University of Edinburgh animal ethics committee and carried out in accordance with the accepted standards of humane animal care under the regulation of the Animal (Scientific Procedures) Act UK 1986 and EU Directive 2010/63/EU. All animals were held in a UK Home Office approved facility. Throughout the project, either wild type (WIK) lines were used or genetically modified lines which were used to explore the imaging of cardiomyopathy (ACM) [17].

### 2.2 Zebrafish MRI system

The MRI system was built in-house using a combination of commercially available components and, where necessary, custom parts which were created in a basic mechanical and electronic workshop. The system is composed of a micro-solenoid radio frequency (RF) coil (internal diameter 6.90 mm) which is integrated into an animal handling system known as a flow cell. The coil is used for both RF signal transmit and receive.

Given the smaller physical size of an adult zebrafish in comparison to rodents, we were able to use a matching small coil with a solenoid geometry for the RF coil to maximize the coil's RF sensitivity [18]. At these dimensions a solenoid geometry is the optimal coil geometry for signal sensitivity, giving up to three times greater sensitivity than a more typical saddle coil of the same dimensions [19]. The flow cell that the subject is placed into was designed to minimize the diameter of the RF coil to allow the greatest coil filling factor, while still holding the zebrafish comfortably in place within the coil. Furthermore, the flow cell also needed to be water tight and to provide a steady supply of oxygenated and anesthetized water around the subject.

### 2.3 Animal handling system

**Fig. 1:**
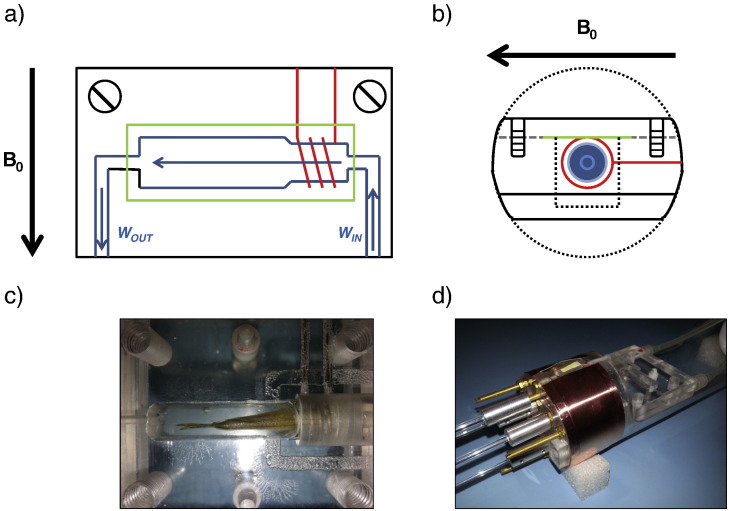
Zebrafish MRI flow cell system – a) top-down view, b) side view. A solenoid coil (red) is integrated with a flow cell, in which a live fish is placed. Water containing anesthesia agent (MS222) (blue arrows) is flowed through the flow chamber (blue) to maintain the fish. The chamber is sealed with a Parafilm gasket (green) sandwiched between the cell and a matching lid (gray dashed line) which is sealed with four screws in each corner (only two shown for clarity), c) photograph of fish in flow cell, positioned for cardiac scanning, d) photograph of the flow cell mated to both holding carriage (transparent) and electronics unit containing the coil circuitry (covered by copper tape for RF shielding) with tuning rods extending from it.

This part of the system consists of an enclosed flow cell to hold the subject in place, an environmental monitoring system to ensure the animal remains physiologically stable during scanning and a flow pump to circulate water continuously through the system. The flow cell is made from three acrylic blocks. The bottom and central blocks are permanently bonded together and the top block serves as a removable lid that is fixed to the lower blocks by four screws positioned at the corners of the block. The flow chamber itself, in which the fish is placed, is machined into the central block of the flow cell. Together, the three blocks form a solid block with total dimensions of 50.0 mm (length) × 57.0 mm (width) × 23.0 mm (height) (see Fig. 1a and b). The flow chamber runs down the central length of this combined acrylic block (Fig. 1c). The channel is narrower at the inflow end to fit more closely around the natural shape of the subject's head which also allows the solenoid coil to be more closely fitted to the subject. The assembly is sealed with a gasket (Parafilm) between the lid and central block to provide a final water tight system that can safely be placed in an MRI scanner.

Water, sourced from aquaria tanks (pH 7, temperature 19–23 °C and aerated), containing a water soluble anesthetic agent (MS222, Sigma-Aldrich (E10521-10G), 100 mg/l) is flowed through the whole system via a Graseby 3150 syringe pump (2.00 ml min^−1^) from a standard 60 ml syringe (BD Plastipak). This provided a constant non-pulsatile flow, which helped to minimize flow artifacts in the MRI images. Additional syringes of MS222 treated water were prepared ready for use, with change as required by the duration of experiment.

The environmental monitoring system was a commercially available system (FireSting O2, Pyroscience). This system was set up to monitor water temperature near the point of entry to the flow cell via a Teflon coated shielded 4-wire PT100 temperature sensor. In addition, water oxygenation levels immediately before and after the flow chamber were monitored via optical sensor probes connected to custom ordered 5 m optical fiber cables, ensuring MRI compatibility. The stability of the local environment was then monitored remotely from outside the magnet room on a computer terminal during scanning. With the difference in water oxygenation, between the inlet and outlet of the cell, giving a real time measure of the oxygen consumption of the fish.

### 2.4 MRI coil configuration

The solenoid micro-imaging coil was constructed from three turns of 0.81 mm diameter susceptibility-matched wire (Cu/Al, Doty), with an internal diameter of 6.90 mm. We found the use of susceptibility-matched wire to be essential given both the small diameter of the coil and the high magnetic field of the MRI scanner being used (7 Tesla). Tests using solenoids made from simple copper wire caused unacceptable reductions in T_2_* relaxation times and gave large susceptibility artifacts in resulting MRI images.

The number of turns was chosen to keep the total length of the solenoid wire small compared to the wavelength of RF radiation used, thereby preventing phase differences between different parts of the coil that would otherwise lead to interference of B1 RF contributions and subsequent signal inhomogeneity. The coil axis was positioned at 90° to the B_0_ scanner field for optimal sensitivity and positioned horizontally so that the zebrafish rested comfortably in the flow cell chamber.

The solenoid was located such that it surrounded the narrower part of the flow cell where the zebrafish head was positioned within (Fig. 1c). The coil was thus in close proximity to the water of the flow cell and the zebrafish, but physically isolated from both, reducing unnecessary loading on the coil and additional capacitances on coil circuitry [20]. This fixed method of coil placement also ensured that the coil position was consistent from subject to subject, helping in the accurate placement of subjects within the scanner.

**Fig. 2:**
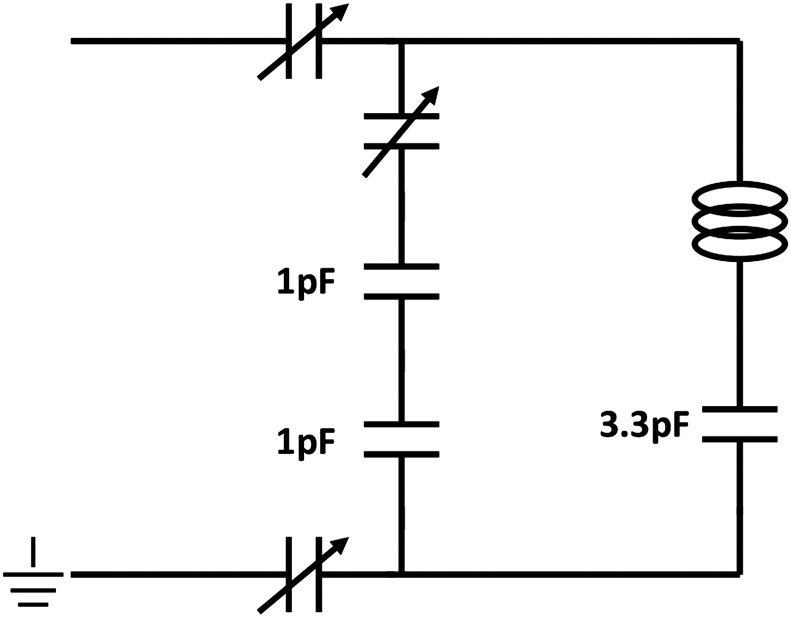
Circuit diagram for the MRI microcoil – the design is a balanced circuit design allowing the coil to be tuned to 300 MHz while fully loaded with flowing water and an adult zebrafish. The variable capacitors have a range of 0.6 pF–9.5 pF.

The RF circuit (Fig. 2) is a balanced coil design [18] which ensured that energy and signal losses due to RF-induced electric fields were minimized. The variable capacitors were non-magnetic and operate in a range of 0.6 pF–9.5 pF (NMQM10GE, Voltronics Corporation) with fixed capacitors as prescribed in Fig. 2 (SQB Low ESR Capacitors, AVX). The circuit was also grounded to help with electronic stability. The circuit housing was surrounded by a strip of copper tape to shield it from RF induced current fluctuations from the MRI scanner during operation.

### 2.5 Subject preparation

Zebrafish (WIK strain) were anesthetized (MS222 125 mg/l) outside of the system and then placed in the flow cell, where they were held in position inside the MRI coil and maintained with a low dose of anesthesia (MS222 100 mg/l). Since anesthetized zebrafish naturally tend to roll onto their backs (ventral surface upward), the anesthetized subjects were supported in an upright position in the flow chamber using small pieces sponge or pieces of paper towel placed laterally between the sides of flow chamber and the fish. The subject was positioned within the coil of the flow chamber, by aligning the eyes with the distal margin of the coil loops. Subsequently, the chamber was sealed. The syringe pump and monitoring equipment were then turned on and flow cell was checked for water leaks.

The whole assembly was then placed inside the MRI scanner. Due to the small linear region of the micro-imaging gradient insert (~3.00 mm parallel to the imaging coil axis), care was taken to position the solenoid in the exact center of the scanner gradient and magnet. Once this had been done the assembly was locked into position via a screw-forced lever system at the top of the assembly.

### 2.6 Imaging acquisition

MRI scanning was performed on a 7T preclinical MRI scanner (Bruker BioSpec) equipped with a microimaging gradient set (1000mTm^−1^, BGA-6, Bruker). Gradient echo (FLASH) and turbo spin-echo (Rapid Acquisition with Refocused Echoes, RARE) based sequences were optimized for structural imaging of the brain and heart.

#### 2.6.1 Phantom scans

MRI coil and image properties were assessed using scans run when the flow cell was filled with a solution of copper sulfate (1 g/l, Bruker Biospin MRI GmbH) using the following parameters:

TR=6000ms,TE=11.7ms,1average,RARE factor=1,matrix=256×256,field of view=1.00cm×1.00cm,number of slices=1,slice thickness=0.50mm,bandwidth=50.0kHz.

**Fig. 3:**
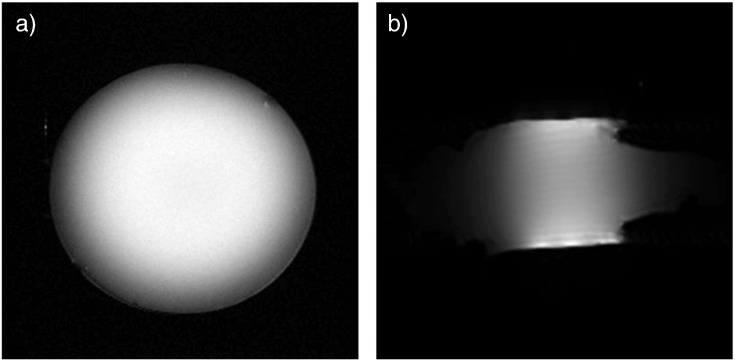
RARE T_2_ images used in coil property measurements – a) perpendicular to the direction of the coil, presenting a smooth round appearance, b) along the axis of the coil showing the shape of the flow chamber itself. Water enters the chamber through the narrow channel on the right.

**Fig. 4:**
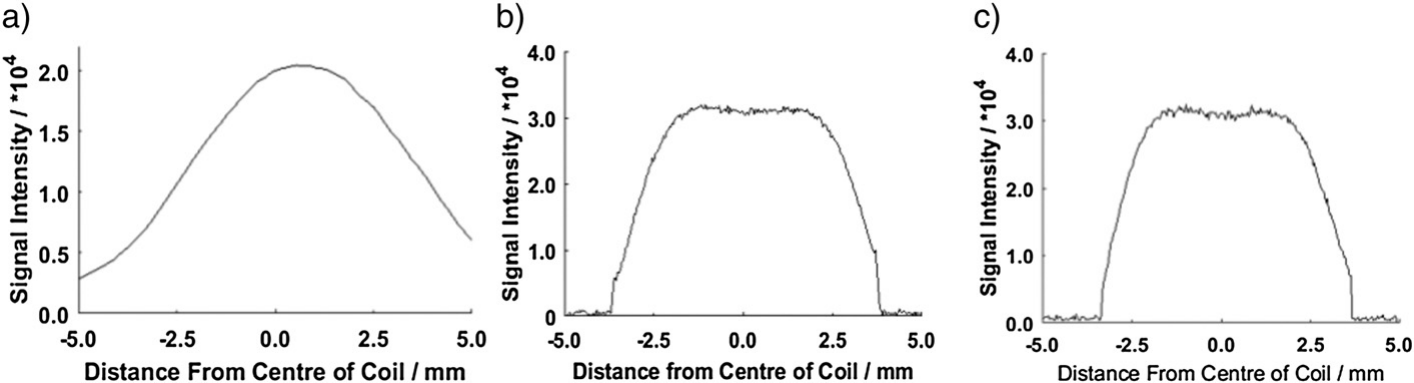
Signal intensity plots showing coil linearity from RARE T_2_ images – a) plot along the parallel axis of the flow cell and coil, b) plot perpendicular to the x-axis of coil cross section, c) plot perpendicular to the y-axis of coil cross section.

Resultant RARE T_2_ weight phantom image slices are shown in Fig. 3, with profiles shown in Fig. 4.

#### 2.6.2 In vivo scans

Two main sets of images were obtained from live subjects. A lower resolution gradient echo (GE) FLASH set of images and a higher resolution RARE T_2_-weighted set.

**Fig. 5:**
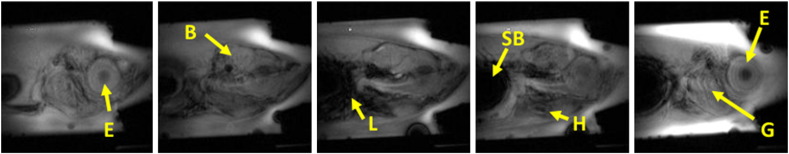
Example in vivo zebrafish MRI images generated using the equipment and protocols outlined in this paper using a Sagittal FLASH-based scan. B = brain, E = eye, G = gill, H = heart, L = liver, M = mouth, SB = swim bladder. Scale bar = 1 mm.

The GE scans (Fig. 5a) were performed at the beginning of each imaging experiment to provide initial images for prescribing subsequent image stacks onto (TR = 100 ms, TE = 3.9 ms, 4 averages, number of slices = 5, slice thickness = 0.50 mm, interslice distance = 0.50 mm, bandwidth = 30.1 kHz, matrix = 170 × 170, field of view = 1.00 cm × 1.00 cm, giving an in-plane resolution of 59 μm and an acquisition time of 48 s).

**Fig. 6:**
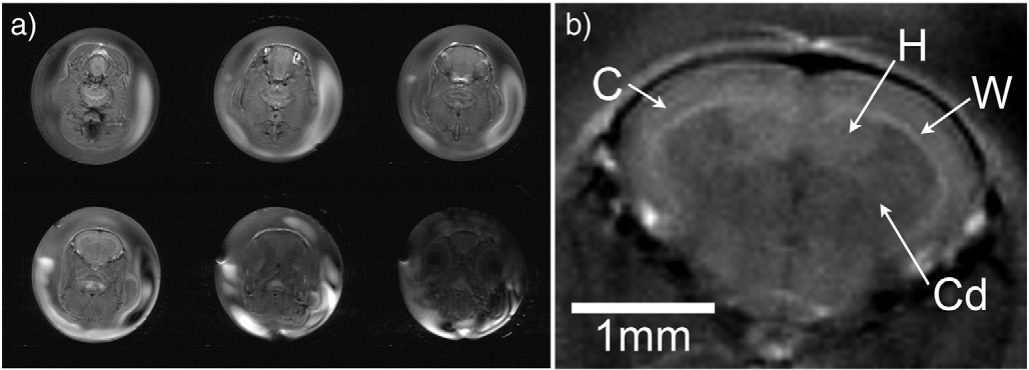
High resolution T_2_ weighted RARE brain MRI, in-plane resolution 39 μm and slice thickness 300 μm. Panel a) shows the 6 coronal slices acquired. b) Shows zoomed-in T_2_ weight image of the brain, with anatomical structures visible; C = cortex, W = white matter tract, H = hippocampus, Cd = Caudate; scale bar = 1 mm.

High resolution multi-slice brain imaging (Fig. 6) was performed on subjects using a T_2_-weighted RARE imaging sequence (TR = 2000 ms, effective echo time = 23.5 ms (echo spacing = 11.7 ms), 10 averages, RARE factor = 4, number of slices = 6, slice thickness = 0.30 mm, interslice distance = 0.60 mm, bandwidth = 50.0 kHz, matrix = 256 × 256, field of view = 1.00 cm × 1.00 cm, giving an in plane resolution of 39 μm and acquisition time of 21 min, 20s).

## 3 Results

### 3.1 Coil properties

The coil Q factor was measured to be 74.1 when loaded via a network analyzer (Agilent 8712ET), when tuned and matched to the scanner operating frequency of 300.1 MHz and at 50 Ω with the −3 dB bandwidth located at 3 dB below the reference level [21].

The signal to noise ratio (SNR) of the coil on the set of phantom images was determined by measuring the signal from the center of the phantom and dividing this by the standard deviation of a region of ‘noise’ outside of the phantom [22]. This was measured to be 126. Signal linearity was defined as being within ±10% of the mean value taken from the central 2.00 mm of the image and these are summarized in Table 1 for all three orthogonal imaging planes. As would be expected signal intensity drops off rapidly outside the regions bounded by the coil locations (Fig. 5).

### 3.2 In vivo imaging

A variety of pre-existing image sequences were trialed, with MRI parameters optimized for use with zebrafish as described in the methods section. Both GE FLASH (Fig. 5a) and RARE (Fig. 6) sequences produced images with comparable visual quality to those of rodent imaging in broadly equivalent acquisition times despite the higher resolution, and so comparatively lower signal per voxel available, required for imaging of zebrafish [23], [24]. With the RARE sequence giving an SNR of 57.1 in the cortex and 47.9 in the sub-cortex, whereas the FLASH sequence gave an SNR of 56.5 over the whole brain.

Anatomical structures can clearly be identified in the gradient echo images shown in Fig. 5, including the gills (G), eyes (E), brain (B), heart (H), liver (L) and swim bladder (SB).

The high resolution T_2_ weighted brain images show details of the zebrafish brain structure, including cortex, caudate, hippocampus and white matter tracts (Fig. 6). The relative resolution obtained for the zebrafish brains (70 voxels across maximal width of brain) is comparable to that obtained for mice brains (~100 voxels across maximal width). However there is innately less structure to see in zebrafish, as complexity (e.g. percentage of white matter) decreases the smaller the species (e.g. human 60%, 11.6% mice) [25].

“High resolution imaging of live zebrafish within a chamber of flowing water presents challenges to MRI, resulting in image artifacts. Artifact due to the flow of the system water (i.e. bright/dark region), can be seen in Fig. 5, Fig. 6, due to both in flow of unsaturated water into the imaging slice and the effect on image encoding. These artifacts disappear when the flow is turned off and were reduced by using a non-pulsatile pump to circulate the system water. Artifacts due to the motion of the fish were initially seen, though this was much improved by finding the correct level of water soluble anesthetic. In addition, the air tissue interface around the air bladder of the fish results in susceptibility artifacts on the gradient echo, see Fig. 5a. Occasionally small air bubbles in the circulating water also produced localized susceptibility artifacts near the surface of the fish.”

### 3.3 Subject welfare/response

While there was some variability in the length of time it took for induction of anesthesia, most subjects were successfully anesthetized within 2–3 min. The depth of anesthesia was assessed by ataxia, propensity of subject to rotate onto their back, non-responsiveness to touch stimuli and visual observations of decreased movement of the operculum (gills). In general, larger fish took longer to be induced as might be expected although this was not the case for every fish. These observations are consistent with those of the wider zebrafish community where induction and maintenance of anesthesia are well recognized to vary widely between and within background strains.

**Fig. 7:**
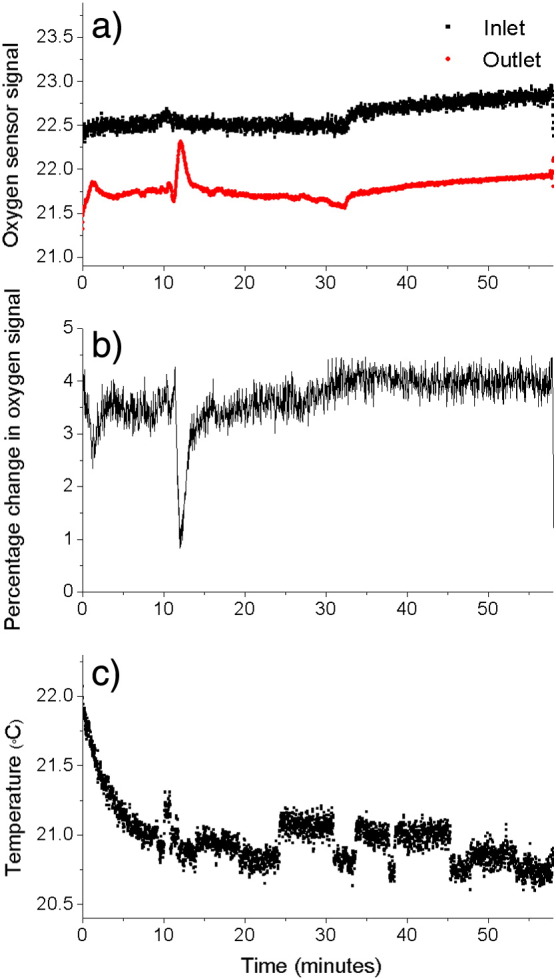
Shows typical recording of the local environmental conditions in the flow cell over a time period of 1 h. a) Shows the water oxygenation at the inlet and outlet. b) Shows the percentage change in oxygenation, indicating oxygen consumed by the fish. C) Shows the local temperature of flow cell water. Note, the spikes at 12 min were due to transfer to MRI.

Scans were obtained over a period of time not exceeding 60 min, as permitted by licensing regulation. An example time series for a typical in vivo MRI experiment is shown in Fig. 7. All scanned subjects showed rapid recovery after scanning was complete when they were immediately placed in normal system-water containing no anesthetic. During recovery, subjects were typically swimming within a few minutes with normal swim behavior resuming within 5–10 min.

## 4 Discussion

We have created a low-cost system for obtaining MRI images from the in vivo adult zebrafish under optimal physiological conditions. Fish scanned under our current in vivo protocols showed no obvious signs of distress or injury following scanning allowing for repeated longitudinal imaging studies.

The usable imaging region obtained using our system is sufficient to encompass the head and pericardial sac of the zebrafish as far back as the front of the first swim bladder. Within this region can be found the brain, heart, liver and respiratory organs allowing each to be imaged at the same time.

To date, the use of MRI techniques in zebrafish has seen limited to a handful of demonstration cases [12], [13], [14], [15], [16]. Among these previous studies the equipment used (both for MRI and for animal maintenance and monitoring) has been crude particularly to physiological maintenance and welfare of the subjects. In addition, these previous in vivo studies have been performed on vertical magnets with the fish in a vertical position. This is a very unnatural position for the fish, particularly over protracted periods and again would add physiological strain. In general, such reports have used pre-existing MRI coils that are not tailored to the species, including the use of expensive cryogenic coils to boost signal reception. Many of these previous studies have been performed using ex vivo or fixed fish [26], [27], [28] as opposed to our system that uses live subjects with full recovery.

To date the highest resolution in vivo T_2_ weighted brain imaging was performed by Kabli et al. [14], who achieved an in-plane resolution of 78 μm with a 0.2 m slice thickness (voxel volume 1.2 × 10^−3^ mm^3^) using a 17.6 Tesla magnet in 8 min. In comparison, the system presented here achieved an in-plane resolution of 39 μm with a 0.3 m slice thickness (voxel volume 0.46 × 10^−3^ mm^3^) using a 7 Tesla magnet in 21 min. Hence, the solenoid RF coil presented here allowed greater spatial resolution to be achieved, using a much lower magnetic field strength.

Previous studies have also failed to promote the wider uptake of zebrafish MRI whereas we believe the quality and comparative speed of the images obtained with our system, combined with its zero mortality rate, provide a significant demonstration that the use of MRI is a viable imaging modality for this species. Given the rapidly increasing pace of research utilizing zebrafish (and other small fish) and the current limited application of other non-invasive imaging techniques for adult zebrafish (such as ultrasound), we believe that zebrafish MRI can make an important and timely contribution across a range of biological fields.

Since there are already several human disease models of biomedical interest that manifest longitudinally in the adult zebrafish, but not in more typical disease models such as rodents or rabbits [29], [30], zebrafish MRI would provide a unique ability to study these diseases non-invasively. Additionally, the zebrafish and other fish are being increasingly used as a model organism in evolutionary biology and the aquaculture industry [31], [32] where MRI could be a novel and useful technique to assess changes in anatomy and physiology of fish under different evolutionary and environmental pressures.

## 5 Conclusions

We have developed and implemented a practical system for in vivo MRI of adult zebrafish. This system integrates a sensitive RF solenoid coil incorporating a physiological flow system. Additionally the system includes remote physiological monitoring of the subjects i.e. temperature, water oxygenation, etc. Animals can be successfully maintained and recovered with image acquisition times of up to one hour trialed. Early studies have suggested that this could even be increased given that the subjects showed no signs of altered behavior or injury upon recovery under these time constraints. The MRI images obtained are of the similar quality to those obtained in rodent MRI studies using similar acquisition times. The inherent soft tissue contrast options available with MRI, combined with MRI's non-ionizing nature could help provide a comprehensive and flexible imaging tool not currently available to the zebrafish community.

## Figures and Tables

**Table 1:** Coil linearity measurements showing good signal quality over required region.

Direction	Linear region from center of image/mm
Perpendicular to coil axis (horizontal)	4.52
Perpendicular to coil axis (vertical)	4.44
Parallel along coil axis	3.06
